# Stakeholder perspectives on the potential benefits, risks, and implications of an interactive cognitive monitoring app for the identification and monitoring of cognitive decline in adults at risk of dementia

**DOI:** 10.1186/s12913-026-14613-z

**Published:** 2026-05-11

**Authors:** Daim Syukriyah, Claire Hulme, Chris Davis, David J Llewellyn, Louise Allan, Dag Aarsland, Clive Ballard, Chris Fox, Sarah E. Lamb, Anne Corbett

**Affiliations:** 1 Department of Health & Community Sciences, University of Exeter Medical School, University of Exeter, Exeter, UK; 2 Centre for Healthy Brain Ageing, Institute of Psychiatry, Psychology & Neuroscience, King’s College London, London, UK; 3 Department of Clinical & Biomedical Sciences, University of Exeter Medical School, University of Exeter, Exeter, UK; 4 Department of Public Health and Sports Sciences, University of Exeter Medical School, University of Exeter, Exeter, UK

**Keywords:** Mild cognitive impairment, Pre-dementia, Dementia, Alzheimer’s disease, Digital cognitive assessment, Cognitive monitoring, Health services research, Implementation, Stakeholder perspectives, Qualitative research

## Abstract

**Background:**

Computerised cognitive testing offers potential for large-scale cognitive assessment and may support the identification and monitoring of early cognitive impairment, while reducing clinical burden on healthcare staff and improving efficiency.

**Methods:**

Three focus groups, comprising members of the public, clinicians, commissioners, business leaders, and academics, were conducted using a semi-structured interview guide addressing acceptability, engagement, benefits, risks, and adoption. Data were analysed using thematic analysis.

**Results:**

Participants identified that the successful implementation of an interactive cognitive monitoring app would depend on its acceptability, perceived usefulness, sustained engagement, accuracy and appropriate interpretation of information, robust data security, and potential efficiency gains within the health and care systems. Perceived benefits for individuals include reassurance, reduced stress, early access to assessment and support, as well as the potential to maintain quality of life. For clinicians and the health system, perceived benefits include improved efficiency through reduced consultation times and shorter waiting lists.

**Conclusions:**

The findings align with existing literature on earlier identification of cognitive decline and digital cognitive assessments, highlighting the potential relevance of such technologies for supporting service delivery and enhancing efficiency within cognitive health care.

**Trial Registration:**

Not applicable.

**Supplementary Information:**

The online version contains supplementary material available at 10.1186/s12913-026-14613-z.

## Introduction

Mild Cognitive Impairment (MCI) affects 10–20% of older adults, 10–15% of whom will develop dementia each year [1, 2]. Individuals with MCI exhibit symptoms including issues with memory, language or thinking compared to their past performance. Symptoms are subtle, and individuals are still able to perform their daily activities without any interference [3, 4]. A minority with MCI receive a diagnosis but are often left unsupported and unmonitored until their condition worsens; 99% of people with MCI never receive a diagnosis, and do not reach the health services until their cognition declines [5]. There is growing interest opportunities to delay or reduce the risk of dementia is considerable including disease-modifying treatments. There is also evidence for health and lifestyle factors that influence prognosis and risk, and for lifestyle interventions that maintain cognition in the early stages [6]. Cognitive training offers a low-risk, low-cost, effective means of maintaining cognition without need for clinician support. However, opportunities are not being realised due to lack of sensitive, accurate cognitive assessment and monitoring in early stages and lack of means of keeping patients engaged with their healthcare and cognitive health from home. This contributes to the public health crisis posed by dementia, which threatens to overburden primary care services. It also limits clinicians’ ability to provide personalised care such as precision medicine, and to support to individuals in making timely lifestyle changes.

Computerised cognitive testing offers potential for population-level cognitive assessments; and an opportunity to support the identification and monitoring of early cognitive impairment, whilst driving efficiency, filling gaps in clinical pathways, and reducing assessment burden on healthcare staff. Whilst there are several technologies in late-stage development, they mostly lack longitudinal validation data supporting the identification of cognitive decline, evidence of sustained long-term user engagement, and the capacity to support remote testing without clinical supervision. Clarity is needed regarding the extent to which these technologies can support the identification and monitoring of cognitive change, including progression from subjective and pre-clinical impairments to early dementia states.

REACTIVE, a combined technology, brings together an evidence-based cognitive training intervention, REACT, with a computerised neuropsychology battery, the PROTECT cognitive test system, into one user-friendly patient-facing app that is designed to deliver digestible, relevant data to clinicians to support the cognitive monitoring and treatment pathways in NHS settings in the UK. This work aligns with recent work highlighting the potential value, effectiveness and acceptability of digital health solutions for older adults and individuals with cognitive impairment [7–9]. These technologies have been successfully used in research, but it is unclear how they would translate to use in real-world settings for the purpose of cognitive monitoring. It is important to understand the relative risks and benefits associated with this type of technology prior to embedding it in clinical practice, and to explore potential impacts on the health and care sectors, clinicians and patients as well as any potential hurdles for its adoption.

This paper explores key stakeholders’ perspectives on potential benefits, risks and implications of use of computerised cognitive testing in an interactive cognitive monitoring app, to support the detection and monitoring of clinically meaningful cognitive decline in adults at risk of dementia.

## Methods

This qualitative study used three focus groups with key stakeholders; the first group consisting of members of the public; the second, clinicians and commissioners; and the third, business leaders and academics. Focus groups were chosen to identify a range of different views and to understand the issues from the perspective of the participants themselves [10].

The study aimed to recruit six to seven participants aged ≥ 18 years to each group. Recruitment to the group for members of the public was through the PROTECT study [11]; an online platform with an active cohort of 30,000 older adults. PROTECT has full consent for recontact and a track record of success in recruiting participants to a wide portfolio of research, including qualitative studies. Participants were invited to take part by email using the established PROTECT study communications library. Clinicians/commissioners were recruited through clinical networks held by the ReaCTIVE study investigators. Those approaches included specialist and generalist clinicians and NHS commissioners. Similarly, business leaders/academics working in relevant fields were recruited through investigator networks and the University’s Exeter Innovation team. For all groups, those expressing an interest were contacted by email by a researcher with details of the study including the participant information sheet (PIS) and consent form. Consent was in place prior to each focus group. All participants had the opportunity to talk to the researchers prior to consent to participate. Participants could withdraw at any time without any consequences.

The focus groups were held online via Microsoft Teams, using a semi structured topic guide and chaired by a member of the research team. An overview of the study context was provided as an introduction. Focus groups lasted up to 60 min, were recorded and subsequently transcribed. All data were anonymised. The semi-structured interview schedule contained questions on acceptability, engagement, benefits, risks, other impacts, and adoption. Qualitative data from focus groups were analysed using thematic analysis, with initial coding informed by the focus group topic guide and used to examine patterns within and across topics [12]. The focus group topic guide was developed by the research team specifically for this study (see Supplementary File 1).

Ethics approval was received from the University of Exeter, Faculty of Health & Life Sciences Ethics Committee on 12th December 2024.

## Results

The focus groups were held in March/April 2025 with 15 participants across the three groups; six attended the members of the public group (*n* = 3 male, *n* = 3 female), five the clinician/commissioners group (*n* = 3 GPs, *n* = 1 secondary care physician, *n* = 1 commissioner; *n* = 3 male, *n* = 2 female) and four business leaders/academics (*n* = 3 business leaders (two of whom also held academic roles) and *n* = 1 GP (who was unable to make the date for the clinician focus group); *n* = 3 male, *n* = 1 female). Across the groups there was wide geographic representation including England, Scotland and Wales. The duration of each focus group was 45–60 min. Results from the three focus groups are presented together (below) as the groups identified similar themes.

### Acceptability

Participants were generally positive. The app would be welcome and could help reduce clinicians’ workloads. Participants thought that it would enable individuals to monitor their own cognitive health performance and minimise unnecessary in-person visits to clinicians. The evidence-based nature of the app would be welcomed by clinicians.

This view was reflected in participants’ own statements: “I think it could be really useful… I’d like to know how much I’m deteriorating… Those of us who want to know will use it, and other people who want to pretend it’s not happening won’t use it“ (Public participant), and “It sounds fantastic… having something more objective that can measure over time… The concern is what we then do with that information, particularly given limited capacity in services“ (Clinician).

There was also discussion around whether the app would be universally accepted by different sub-sets of the population (or potential users). It was posited that those who have concerns about their brain health are more likely to use it. Yet, for those who are more fearful of decline or who do not think that decline is happening, they would be less likely to welcome the app and might need to be encouraged to use it.

Socio-demographics were thought to be important, suggesting that people who live in wealthier areas would be more likely to accept and use the app with greater adherence than those living in less affluent areas. Education was also thought to play a role, particularly in relation to health literacy, as was age. The younger generation might be more reluctant to seek information about their brain health or to engage in follow-up assessment, while older people tend to be receptive to cognitive assessment and monitoring of their brain health. For example: “I suspect we’d find a massive health inequality in uptake… in more affluent areas people would do this diligently, but in deprived areas uptake may be lower“ (Clinician).

There were, however, questions around whether older people are less technologically literate than younger groups, although the move towards universal ownership of mobile devices suggests this viewpoint may be losing traction. Overall, participants felt that there could be inequality in uptake among different population groups, for example by age or income. The quality of the device used to access the app, as well as the app interface itself, was thought to be influential in whether the app would be welcomed. For example, answering questions using a computer does not provide the same experience as using a mobile device, given the wide variation in device quality. Thus, not only do users need to be at least familiar with using apps, but the quality of the app interface and the device used also matter in supporting access to the app features.

### Engagement

Participants felt that the benefits of the app would depend on engagement over time, which in part would be influenced by its perceived usefulness. In addition, it should be fun to use and tailored for the individual. Participants also thought that feedback and prompts are important for maintaining engagement. Users who remain interested in their cognitive health would be more likely to continue using the app if prompted. If the app can identify changes in cognitive function at an appropriate time, people with MCI might benefit from earlier access to support and treatment. It is key to remind people to keep using the app if the app can provide an update via scoring (and their scores in relation to their peers) so a person can track their progress on their cognitive health over time.

Those views are illustrated in participants’ responses: “We do a full cognitive assessment… it takes nearly 25 minutes, and I get bored doing it… a lot of users don’t complete it… we’ve reduced it to around 8 minutes because that’s what people are prepared to do” (Business/academic participant), and “We will use the app… as long as we’re prompted to do it… letting us know how our score changes over time is important” (Public participant).

Tailoring the app to the user was also seen as important. Participants noted that individual preferences vary; the app might need to offer basic and premium/advanced app versions to accommodate users who thrive on new challenges, whilst others might find it too complicated to learn new app features or information with the updated version.

Participants suggested that to maintain a long-term user connection, the app not only needs to serve brain stimulation function through games and tests but also act as a tool to promote brain health and encourage improvements in areas such as physical mobility or nutritional intake. The app could also be integrated with other transforming care in later life initiatives to monitor users’ physical frailty, hearing loss, and other areas that have direct interlinkage between those aspects and the risk of dementia. The sharing of preventative information or training, for example to support nutrition or awareness of hydration, was also highlighted.

Involving family members was thought to be important to ensure awareness of the app and its functions. Family members could help remind users within their families to regularly access cognitive brain training and other assessments. Hence, throughout their cognitive journey, the longevity of the app use is maintained. For example: “They needed a lot of support from us… to encourage them to participate… bringing in family or social support may be important to maintain engagement” (Business/academic participant).

### Benefits

The primary benefit was that the app could serve as a structured, data-informed measure of cognitive health, supporting earlier identification and monitoring of cognitive change. The suggested benefits fall broadly to the users, the clinicians, and the wider health system.

Accessibility was seen as a key benefit, with participants noting that users could access the app and its programme in their own time, supporting more timely assessment and monitoring of cognitive health. Participants perceived that the app had the potential to empower users by supporting engagement with their cognitive health, including awareness of changes over time. The data generated by the app could facilitate access to professional review and follow-up assessment where concerning information was identified. For example: “Accessibility is probably the buzzword you could use… very easy for people to access in their own time” (Business/academic participant).

Participants suggested that use of the app could reduce anxiety around MCI and provide reassurance regarding cognitive health related to dementia. They noted that arranging appointments with a clinician or GP can take time for individuals with memory concerns. Using the app may provide reassurance to users if they have no memory problems, or prompt referral to a GP where concerns are identified. The app may offer a greater sense of control over users’ concerns about their cognitive health.

Participants noted that clinicians would receive patients’ brain health information from the app, which could reduce consultation time and enable allocated time to discuss subsequent treatment or follow-up activities. However, they also highlighted concerns that clinicians may not welcome, and could be overloaded by, the passive receipt of continuous cognitive health data within GP records. For example: “If a patient comes with data already, that’s brilliant and helps use consultation time more effectively… but passively receiving continuous data into GP records wouldn’t be appreciated and likely wouldn’t be looked at” (Clinician).

The app could potentially reduce onward unnecessary referrals, which might benefit the NHS and help reduce clinicians’ workloads. It has the potential to make the healthcare system more efficient and reduce waiting times to see clinicians.

### Risks

Three areas of risk were identified: inaccurate information, lack of clinician engagement, and data breaches or cyber security concerns.

Concern was expressed around the possibility of false negatives and false positives. Participants noted that inaccurate results could arise due to factors such as technical issues with the app, user error, or the time of day the app was used. In the case of the latter, the time and frequency of undertaking the tests and exercises can affect the scores and test outcomes. Participants therefore highlighted the need to uniformly determine and inform users about the timing and frequency they access the app and do the exercises.

Inaccurate results and information may generate unnecessary stress for users instead of reassurance. Participants highlighted that false positive results could lead to referrals for further assessment, which may be stressful for users and inefficient for GPs and the NHS. The opposite may also happen, where a user has a cognitive issue, and the app does not pick it up. It was noted that, even in the absence of false negatives or false positives, users may experience anxiety about their results, especially if they face indications of cognitive decline outcomes based on self-assessment. For example: “The only concern I’d have is if there was cognitive decline. Is it a true representation of what’s happening or is there user error… it may cause unnecessary stress and distress for people, the app might have glitched, or they think they were doing something which suddenly causes a big drop in cognitive decline, which isn’t true” (Clinician).

Concerns were raised that, while the app has the potential to support earlier identification and monitoring of cognitive concerns, clinicians’ preferences may vary regarding information conveyed and their perspective on its usefulness. For example, some clinicians may not trust the results generated by the app. It was also suggested that use of the app could increase demand on healthcare services and therefore contribute to additional financial burden for the healthcare system.

It was noted that GP practices can vary hugely in terms of how cooperative and responsive they are to patients, which may contribute to inequalities in service provision. Participants highlighted that, even if the app is used to identify changes in brain function, GPs and clinicians remain responsible for reviewing the information received, and conducting further assessments, which may require additional time and resources.

Participants also expressed concerns about the potential risk of data breaches, where information derived from the app data could be misused. They emphasised the importance of ensuring that data are used appropriately for the purpose of identifying changes in cognitive function, with clear safeguards to prevent unauthorised access or inappropriate sharing. It was suggested that, where possible, integration with existing NHS systems, such as the NHS App, could help address these concerns. For example: “I would just give some general risks with any app. One would be patient identification. This is very sensitive information… if there was a data breach, that would probably be the end of this app… somebody could exploit that information about vulnerable people” (Clinician).

### Impacts

Other impacts discussed were associated primarily with early referral. Participants considered that the app has the potential to facilitate earlier identification of dementia-related concerns, which may enable earlier access to support and treatment. This was felt to have impact on mental health of the patients, which subsequently may also reduce the burden of the mental health services in the healthcare system. People often delay checking cognitive decline issues, and the app may enable people to become more informed about their cognitive health and attend the GP surgery when concerns are flagged in the system. This could drive more attendance if uptake is high.

### Adoption

Hurdles to adoption by the health system were discussed, including public resistance, cost to the NHS, and competition from other digital tools. Public resistance centred around two key issues. The first related to how users’ data might be used. Detailed personal information gathered from users’ needs to be used appropriately. The members of the public group felt data from users should not be used for commercial activities and to generate monetary benefits for certain parties (‘shareholders’ pockets’). For example: “There’ll be some people… who are concerned that their data is used for commercial purposes… they would want reassurance that it’s used for research rather than for lining their pockets or their shareholders’ pockets” (Public participant).

The second point focussed on addressing public resistance through the need for champions and a clear strategy to promote the benefits of using the app. For example, participants suggested that relevant leaders or individuals running clinics could promote and advocate the use and advantages of the app, especially for people who have concerns about their cognitive health or general brain health.

Cost to the NHS was also identified as a hurdle to adoption, given existing budget constraints and the challenge of asking patients to pay for access. While the app was seen as having the potential to improve efficiency, it was noted that increased uptake could lead to higher demand for services and have consequences for workforce capacity and costs. The costs associated with procurement, maintenance, and running the operation, as well as the process of adoption within the NHS system, were also seen as potential challenges.

Competition with other similar tools or apps in the wider commercial market means that the app needs to position itself clearly in terms of what it offers that differentiates it from similar existing options in the market that makes people willing to invest in using it.

## Discussion

The results of this study have resonance with existing literature both around earlier identification of cognitive decline and digital cognitive assessments. In line with previous research, potential benefits for individuals include access to therapies, services, agencies and support networks, which may facilitate early adaptation, early coordination with care plans and organisation of support [13–16] There may also be potential benefits in delaying or mitigating progression of cognitive decline, for example through modification of exposure to particular risk factors [13, 14]. Early identification may alleviate worry and reduce anxiety about symptoms [15, 16], and provide individuals with a sense of control, agency, or empowerment over their condition. This has been associated with maintaining independence in their own home and quality of life for longer when support is provided early [15, 16]. Early identification can allow opportunities to plan ahead and participate in legal, financial, care options, or other wishes to their family members [15, 16]. Over time, individuals may be able to track their cognitive health, potentially increasing commitment to their wellbeing [17].

Conversely, early identification may lead to fear, anxiety, depression, stigma and altered relationships for both individuals and their caregivers [13, 16]. Individuals may feel anxious due to the stigma of Alzheimer’s disease (AD) and vulnerable due to uncertainty around the extent and timing of deterioration; this may increase the risk of withdrawal, isolation, and social exclusion for both individuals and caregivers [13, 14]. Whilst stigma was not explicitly mentioned by participants in the focus groups, it was noted that there may be anxiety about the results, particularly when facing the negative findings or indications of cognitive decline outcomes based on self-assessment. Dubois and colleagues note that cultural beliefs can influence the degree of perceived stigma [16]. Focus group participants suggested that socio-demographics may play a role in whether the app in this context would be welcomed or accepted. This is important, as disparities in timely diagnosis have been found across race, ethnicities and educational backgrounds [18], and could potentially lead to, or exacerbate, existing inequalities.

Given participants’ concerns regarding inequalities in uptake, particularly among individuals in less affluent areas or with lower digital literacy, targeted engagement strategies may be required to support adoption. This may include clear communication about the purpose and benefits of the app, alongside the use of existing healthcare and community channels to increase awareness and trust. Engagement may also be strengthened through the involvement of trusted individuals, such as clinicians, those overseeing relevant services, or other community-based advocates, alongside proactive outreach and support from health systems or public services to reach individuals from socioeconomically disadvantaged backgrounds.

Evidence from systematic reviews of digital health interventions suggests that uptake and engagement are influenced by factors such as accessibility, cost, user awareness, support, and eHealth literacy, highlighting the importance of tailored strategies to promote sustained and equitable use of digital health tools [19, 20]. In line with this, our findings further suggest that the acceptance and adoption of digital cognitive assessment tools are shaped not only by structural factors such as access and cost, but also by trust, perceived risk, and individuals’ capacity to engage with and interpret digital health information.

Additionally, system-level support is important to achieve equitable uptake. This has implications for policymakers, highlighting the need for targeted strategies and a coordinated approach to ensure digital cognitive assessment tools are accessible and beneficial across all population groups. Without such approaches, there is a risk that digital interventions may disproportionately benefit better-off groups, which may worsen existing inequalities in access to early cognitive assessment, monitoring, and support.

Whilst there was less focus group discussion around the benefits to family members and caregivers than has been reported elsewhere, these benefits were implicit in participants’ discussions, including the suggestions that involving family members could be helpful and may support their quality of life. Access or earlier access to appropriate agencies, resources, and support groups has been shown to reduce carer strain and potentially reduce informal care costs [13–16]. Early identification can enable future planning for solutions in care and emotional burden of disease; for care plans to be initiated, facilitating the anticipation of future problems such as risk of falls, financial mismanagement and vulnerability to scams [16]. The provision of additional time to adapt following early identification can help carers to feel less anxious and stressed, improving family and caregivers’ ability to offer care, which may potentially delay institutionalisation in the future [13–16]. However, early identification may also negatively affect the caregivers through increased care burden from an early stage due to hypervigilance, the impact on financial resources and greater need for practical assistance [14].

The benefits to health and social care sectors discussed in this study align with previous research in this area, including reduced and potentially more efficient use of services, with implications for cost reductions [13–15, 21]. For the social care sector, this may arise through delaying institutionalised care, including nursing home or care home admission, as well as the need for home care and respite care [13, 15]. For the health system, earlier identification may enable more effective intervention provision at a less impaired or more functional stage, which has been associated with reduced hospitalization [14, 15]. Early identification may also support the establishment of relationships with family members and caregivers, facilitating the provision of appropriate advice and information and encouraging dialogue across the health sector regarding care and management [16].

Conversely, concerns were expressed in the focus groups that the app could increase demand and financial burden on the healthcare system. Similar challenges are noted in the literature, including limited preventative or treatment options and shortages in specialist diagnostic services [16]. Increased workload for GP services might be anticipated as referrals increase, together with concerns about increased demand due to diagnostic errors, where inaccurate or unnecessary referrals are likely to be stressful for users and inefficient for GP services. Whilst diagnostic errors are of concern, digital technologies may allow for naturalistic data collection that can more closely reflect real world cognitive function and automated scoring can reduce clinician time and likelihood of scoring errors [17, 21]. This aligns with the rationale for the wider REACTIVE research programme, which aims to evaluate the role of digital cognitive monitoring tools within clinical pathways, including their potential to support identification and monitoring of cognitive change within primary care settings. This finding also supports the rationale of the programme to run a full-scale clinical investigation to establish the impact on service use and engagement with primary care as a measure of impact on NHS efficiency and burden.

There were also questions around the cost of computerised cognitive testing systems, such as REACTIVE, to the health system given limited budgets and competing priorities. Any such system would need to be efficient and cost-effective to be adopted by a health system. This would, in part, depend on acceptance of, and engagement with, the technology. Acceptance may depend on factors including the users’ attitudes to their brain health, the quality of the device used to access the app, the app interface, and familiarity with technology. The move towards universal ownership of mobile devices suggests that familiarity with digital testing may be less of a concern than previously. Whilst some evidence suggests no difference in computerised cognitive test scores at group level between older adults with and without technology experience, further research is needed [22]. From the clinician’s perspective, engagement, automated delivery, and easy-to-interpret results in a safe way are key to clinical utility [22].

These findings highlight the importance of careful integration of digital cognitive assessment tools within existing clinical pathways, ensuring that potential increases in demand are balanced against gains in efficiency, cost-effectiveness, and clinical utility, without placing unintended strain on primary care services. For technology developers, this highlights the need to design apps that are easy to use, provide clear explanations of results, and help users feel confident and comfortable using them over time.

Further research is needed to examine the acceptability and uptake of digital cognitive assessment tools across diverse populations, particularly among groups at risk of digital exclusion. Longitudinal studies would help to understand sustained engagement over time and whether these tools can support ongoing cognitive monitoring and early identification in real-world settings. Comparative studies with traditional cognitive assessment approaches would also be valuable in establishing their relative effectiveness and role within clinical pathways.

### Strengths & weaknesses

Previous work in early detection and/or prediction of dementia has focussed on cognitive assessment in clinical practice (see for example [13, 14]), with calls for the use of digital cognitive assessments for dementia gaining traction [21]. However, there is a scarcity of implementation studies of scalable self-administered instruments which could identify facilitators and barriers to clinical applications [22]. This study adds to this evidence base, exploring the potential benefits, risks, and implications associated with use of computerised cognitive testing through an interactive cognitive monitoring app, to support the identification and monitoring of clinically meaningful cognitive decline in adults at risk of dementia. Specifically, the app is designed for self-completion outside the clinical environment, which has received less attention in the current literature. The findings of this study may help inform the development and refinement of digital cognitive assessment tools, particularly in ensuring that design and data delivery systems align with user needs. They may also provide insights relevant to future implementation, including considerations around scalability, adoption, and potential pathways to integration within health systems and wider deployment.

In terms of the study design, whilst the focus group participants represented all key stakeholder groups, the participant numbers in two groups were slightly lower than planned, which may have impacted the diversity of perspectives captured. In addition, recruitment through the research team’s existing networks, and the inclusion of members of the public who participate in a longitudinal cohort that complete a series of online questionnaires on a yearly basis, may introduce selection bias. However, as highlighted earlier, the findings are consistent with existing literature on early identification of cognitive decline and digital cognitive assessments.

## Conclusion

Computerised cognitive testing offers the potential for affordable, widespread cognitive assessment and the opportunity to support the identification and monitoring of cognitive impairment, as well as earlier access to assessment and care. The potential benefits, risks, and implications of using these technologies are wide-ranging for individuals and their families or caregivers, clinicians, and the wider health and social care sectors. Perceived benefits for individuals include reassurance, reduced stress, and earlier access to assessment and support, as well as the potential to maintain quality of life. For primary and secondary care clinicians and the health system, potential benefits were perceived to include improved efficiency, for example through reduced consultation times and shorter waiting lists. The successful implementation of an interactive cognitive monitoring app will depend on factors including acceptability and perceived usefulness, sustained engagement, accuracy and appropriate interpretation of information, minimisation of data security risks, and evidence of improved efficiency within the health and care systems.

## Electronic Supplementary Material

Below is the link to the electronic supplementary material.

Supplementary Material 1

## Data Availability

The datasets generated during the current study are not publicly available due to the qualitative nature of the data and the potential for participant identification. In line with ethical approval, identifiable data and anonymised transcripts were securely deleted following analysis, and no datasets are available for sharing.
