# Supplementary material for: Stakeholder perspectives on the potential benefits, risks, and implications of an interactive cognitive monitoring app for the identification and monitoring of cognitive decline in adults at risk of dementia
Source: BMC Health Serv Res. 2026 May 11;26:919. doi: 10.1186/s12913-026-14613-z (PMC13339268; doi:10.1186/s12913-026-14613-z)
Supplement: Supplementary file 1 — Supplementary Material 1 [file 12913_2026_14613_MOESM1_ESM.docx]

**Working group activity questions**

1. Do you think this type of app used in this context would be acceptable?

*(Prompts): By …*

- Individual
- Clinicians
- Healthcare system
- Other

1. What do you think the potential benefits conferred by this ReaCTIVE app?

*(Prompts): For …*

- Individual
- Clinicians
- Healthcare system
- Other

1. What do you think any potential risk arising from putting in practice of this app?

*(Prompts): For …*

- Individual
- Clinicians
- Healthcare system
- Other

1. What are the impacts of using this app?

*(Prompts): For …*

- Individual
- Clinicians
- Healthcare system
- Other

1. From a commercial viewpoint, what hurdles would be encountered in adoption in the NHS – how can they be overcome/addressed?
